# Anti-proliferative but not anti-angiogenic tyrosine kinase inhibitors enrich for cancer stem cells in soft tissue sarcoma

**DOI:** 10.1186/1471-2407-14-756

**Published:** 2014-10-10

**Authors:** Robert J Canter, Erik Ames, Stephanie Mac, Steven K Grossenbacher, Mingyi Chen, Chin-Shang Li, Dariusz Borys, Rachel C Smith, Joe Tellez, Thomas J Sayers, Arta M Monjazeb, William J Murphy

**Affiliations:** Department of Surgery, Division of Surgical Oncology, University of California Davis Medical Center, 4501 X Street, Sacramento, CA 95817 USA; Laboratory of Cancer Immunology, Department of Dermatology, University of California Davis Medical Center, Sacramento, CA 95817 USA; Department of Pathology and Laboratory Medicine, University of California Davis Medical Center, Sacramento, CA 95817 USA; Division of Biostatistics, Department of Public Health Sciences, University of California Davis, Sacramento, CA 95817 USA; Cancer and Inflammation Program, Leidos Biomedical Research, Inc, Frederick National Laboratory, Frederick, Maryland 21702 USA; Department of Radiation Oncology, University of California Davis Medical Center, Sacramento, CA 95817 USA; Department of Dermatology, University of California Davis Medical Center, Sacramento, CA 95817 USA

**Keywords:** Soft tissue sarcoma, Cancer stem cells, Tyrosine kinase inhibitors, Sorafenib, Pazopanib, Regorafenib, ALDH

## Abstract

**Background:**

Increasing studies implicate cancer stem cells (CSCs) as the source of resistance and relapse following conventional cytotoxic therapies. Few studies have examined the response of CSCs to targeted therapies, such as tyrosine kinase inhibitors (TKIs). We hypothesized that TKIs would have differential effects on CSC populations depending on their mechanism of action (anti-proliferative vs. anti-angiogenic).

**Methods:**

We exposed human sarcoma cell lines to sorafenib, regorafenib, and pazopanib and assessed cell viability and expression of CSC markers (ALDH, CD24, CD44, and CD133). We evaluated survival and CSC phenotype in mice harboring sarcoma metastases after TKI therapy. We exposed dissociated primary sarcoma tumors to sorafenib, regorafenib, and pazopanib, and we used tissue microarray (TMA) and primary sarcoma samples to evaluate the frequency and intensity of CSC markers after neoadjuvant therapy with sorafenib and pazopanib. Parametric and non-parametric statistical analyses were performed as appropriate.

**Results:**

After functionally validating the CSC phenotype of ALDH^bright^ sarcoma cells, we observed that sorafenib and regorafenib were cytotoxic to sarcoma cell lines (P < 0.05), with a corresponding 1.4 – 2.8 fold increase in ALDH^bright^ cells from baseline (P < 0.05). In contrast, we observed negligible effects on viability and CSC sub-populations with pazopanib. At low doses, there was progressive CSC enrichment in vitro after longer term exposure to sorafenib although the anti-proliferative effects were attenuated. In vivo, sorafenib improved median survival by 11 days (P < 0.05), but enriched ALDH^bright^ cells 2.5 – 2.8 fold (P < 0.05). Analysis of primary human sarcoma samples revealed direct cytotoxicity following exposure to sorafenib and regorafenib with a corresponding increase in ALDH^bright^ cells (P < 0.05). Again, negligible effects from pazopanib were observed. TMA analysis of archived specimens from sarcoma patients treated with sorafenib demonstrated significant enrichment for ALDH^bright^ cells in the post-treatment resection specimen (P < 0.05), whereas clinical specimens obtained longitudinally from a patient treated with pazopanib showed no enrichment for ALDH^bright^ cells (P > 0.05).

**Conclusions:**

Anti-proliferative TKIs appear to enrich for sarcoma CSCs while anti-angiogenic TKIs do not. The rational selection of targeted therapies for sarcoma patients may benefit from an awareness of the differential impact of TKIs on CSC populations.

**Electronic supplementary material:**

The online version of this article (doi:10.1186/1471-2407-14-756) contains supplementary material, which is available to authorized users.

## Background

The cancer stem cell (CSC) hypothesis postulates that CSCs, also referred to as tumor-initiating cells, represent a small proportion of malignant cells in the overall tumor bulk [1, 2]. It is these typically quiescent cells which are resistant to conventional cytotoxic cancer therapies and which are able to repopulate tumors even after apparent complete response to chemotherapy and/or radiotherapy (RT) [3–5]. The presence of CSC subpopulations has been identified in nearly all human malignancies, and mounting studies of CSC engraftment in long term culture and immune-compromised mice have validated the CSC phenotype [6–8]. Moreover, genetic lineage tracing studies have provided provocative evidence for the existence of CSCs in a hierarchy of asymmetric cell division and tumor repopulation in models of squamous cell carcinoma, intestinal adenomas, and GBM. These studies provide the highest level evidence to date that CSCs are clinically and biologically significant [3, 9, 10].

Numerous CSC markers have been identified and characterized, including cell surface markers such as CD24, CD44, and CD133, and the intracellular enzyme aldehyde dehydrogenase (ALDH), among others [1, 8, 11, 12]. Although investigators have observed the expression of CSC markers to vary depending on experimental conditions and tumor type, ALDH has been consistently identified as a CSC marker in breast cancer and prostate cancer, and levels of ALDH^bright^ cells have been observed to predict worse oncologic outcome in numerous human cancers, including soft tissue sarcoma (STS) [7, 13–18]. Awad et al., for example, identified an ALDH^bright^ subpopulation of Ewing’s sarcoma cells which was able to stimulate long term colony outgrowth, form tumor xenografts in immunodeficient mice, and resist chemotherapy treatment [19].

Although CSCs are considered resistant to standard anti-cancer therapies such as chemotherapy and RT, few studies have examined the effects of tyrosine kinase inhibitors (TKIs) on CSCs, particularly the differential effects of TKIs depending on their mechanism of action. Sorafenib is a pleotropic TKI which exerts its activity primarily by direct effects on cell proliferation via inhibition of C-Raf and B-Raf [20]. Sorafenib is FDA-approved for the treatment of advanced renal, liver, and thyroid cancer [21], and Phase II studies of sorafenib have demonstrated activity and clinical benefit for patients with metastatic STS [22, 23]. A recently completed Phase I trial demonstrated safety and preliminary data for activity in locally advanced extremity STS [24]. Regorafenib is a second generation multi-kinase inhibitor with activity and mechanism of action similar to sorafenib [25]. Regorafenib is approved for the treatment of metastatic colon cancer and advanced gastrointestinal stromal tumors [26]. In contrast, pazopanib is a potent inhibitor of angiogenesis [27]. In a recently completed international phase III trial for patients with metastatic STS, pazopanib reduced the risk of tumor progression or death approximately 70%, leading to its approval for STS by the FDA in 2013 [28]. Pazopanib is also approved for the treatment of patients with advanced renal cell carcinoma [29].

Given the increasing clinical use of targeted therapies such as TKIs in clinical oncology including STS as well as the evidence suggesting that specific tyrosine kinases may promote the CSC phenotype [30], we sought to determine the effects of TKIs on whole tumor bulk and CSC populations in diverse models of STS. We hypothesized that there would be differential effects of TKIs on CSC populations depending on their mechanism of action and that enrichment for sarcoma CSCs would be more prevalent with anti-proliferative TKIs rather than anti-angiogenic TKIs.

## Methods

### Tumor cell lines

Human sarcoma cell lines (A673 Ewing’s sarcoma, SW-982 synovial sarcoma, and SK-LMS leiomyosarcoma) were obtained from the American Type Culture Collection (Manassas, VA) and maintained in the recommended tissue culture medium supplemented with 10% heat-inactivated fetal calf serum, L-glutamine, penicillin G, streptomycin, amphotericin, and gentamycin.

### Materials

Sorafenib p-Tosylate salt, regorafenib, and pazopanib free base were obtained from LC Laboratories (Woburn, MA). For in vitro experiments, compounds were dissolved in a stock solution of 100% DMSO and then diluted to final concentration of 0.2% DMSO. Stock solutions were replenished every 4–6 weeks per manufacturer’s recommendations. For in vivo experiments, sorafenib was protected from light, dissolved in 10% DMSO, 10% cremaphor, and 80% sterile PBS, and sterile-filtered through 0.2 μM pores (Cole-Parmer, Chicago, IL). Daily intraperitoneal (i.p.) injections were administered using fresh sorafenib. Placebo animals received i.p. injections containing 10% DMSO, 10% cremaphor, and 80% sterile PBS.

### ALDEFLUOR™ assay and flow cytometry

ALDEFLUOR™ expression (STEMCELL Technologies, Vancouver, BC, Canada) was determined according to the manufacturers’ instructions using diethylaminobenzaldehyde (DEAB) to inhibit ALDH activity and to control for background fluorescence (Additional file 1: Figure S1). Pacific Blue anti-human CD45 (HI30) and 7-AAD were purchased from BD Biosciences (San Jose, CA). PE-Cy7 anti-human CD24 and Pacific Blue anti-human CD44 were purchased from BioLegend (San Diego, CA). PE anti-human CD133 was purchased from Miltenyi Biotec (Auburn, CA). All samples were acquired on an LSR Fortessa with HTS (BD Biosciences, San Jose, CA) and analyzed with FlowJo software (TreeStar, Ashland, OR).

### Animals and tumor cell implantation

Female NOD.Cg-*Prkdc*^*scid*^*Il2rg*^*tm1Wjl*^/SzJ (NSG) mice, aged 7 – 8 weeks, were obtained from The Jackson Laboratory (Bar Harbor, ME) and housed under specific pathogen-free conditions. For subcutaneous/flank tumor inoculation, cells were harvested from 80% confluent cell culture conditions, counted, and resuspended in sterile PBS at a concentration of 25 × 10^6^/mL. A total of 5 × 10^6^ cells in 200-μL aliquots were then injected subcutaneously into the dorsal-lateral aspect of the flank. Tumors were allowed to grow to 3 – 5 mm in maximal dimension prior to initiating treatment. For intravenous tumor inoculation, cells were resuspended in sterile PBS at a concentration of 2 × 10^6^/mL, and a total of 2 × 10^6^ cells in 1-mL aliquots were then injected by tail vein. Tumors were allowed to grow for approximately 3 weeks by which time multiple lung and liver metastases were reproducibly visible on necropsy studies prior to initiating treatment. All experimental protocols were approved by the UC Davis Institutional Animal Care and Use Committee.

### Xenograft tumor evaluation

Mice were euthanized at indicated time points. Lung and liver metastases were excised and manually dissociated in PBS to create an homogenous slurry. Collagenase I (1 mg/mL) and DNAase (0.1 mg/mL) were dissolved in 2% BSA (weight/volume), filtered using a 0.22 μM sterile filter (Pall Life Sciences), and mixed with tumor slurry. Samples were incubated at 37^o^ C for one hour, filtered sequentially using 100 μM, 70 μM, and 40 μM filters (BD Biosciences, ), centrifuged at 1200 rpm for 5 minutes, resuspended in PBS, and counted using a Coulter Counter. Repeat centrifugation was performed, and tumor cells were resuspended at PBS at 1 – 2 × 10^6^ cells per mL for flow cytometry.

### Histology and immunohistochemistry

Xenograft tumor samples were fixed for 24 – 48 hours in 10% formalin and then transferred to 90% ethanol. Hematoxylin and eosin (H&E) slides were reviewed in a blinded fashion by a pathologist (M.C./D.B.). Percent histologically intact tumor and percent necrotic tumor were scored per slide, and mean percent tumor necrosis was calculated for the entire specimen, excluding non-neoplastic tissue. Approximately 5 – 10 H & E slides were examined per animal.

### Evaluation of primary sarcoma samples

Primary STS and benign tumor resections (SA-0689, CCS0015-012, CCS0015-010, SA-0624, and SA-0751) were obtained immediately after surgical excision through the UC Davis Comprehensive Cancer Center Biorepository. Informed consent was obtained from all patients before tissue procurement under the auspices of the Institutional Review Board of UC Davis. Primary STS tumor samples were processed into single cell suspensions for CSC phenotyping and ex vivo exposure to TKIs as described above. CD45 negative selection was used to exclude nonneoplastic myeloid and lymphoid cells from analysis, and 7AAD viability dye was used to exclude dead cells.

### Evaluation of archived clinical sarcoma samples

Tissue microarrays (TMA) were constructed using formalin-fixed, paraffin-embedded clinical sarcoma specimens obtained by the UC Davis Cancer Center Biorepository (CCBR) Core Facility. Eight patients were previously treated on a phase I clinical trial protocol (UCDCC#216, NCT) using neoadjuvant sorafenib and RT for locally advanced extremity STS prior to resection with curative intent [24]. Eight STS patients who were treated with primary surgical resection (without neoadjuvant sorafenib and RT) were used as controls. IRB approval for this retrospective analysis of prospectively collected STS tumor tissue was obtained from the Institutional Review Board of UC Davis.

Following antigen retrieval and blocking, TMA sections (4 μm) were immunostained using a commercially-available purified mouse anti-human ALDH1 antibody (BD Transduction Laboratories, San Jose, CA) with appropriate positive and negative controls. We used the avidin–biotin complex method (DAKO) with 3,3’-diaminobenzidine (DAB) for visualization. Stained slides were reviewed by a pathologist (M.C.) who was blinded to the clinical outcome and scored for percentage and intensity of ALDH1-positive cells. The product of the percentage of cells staining positive and the staining intensity was then calculated as described previously.

### Statistical considerations

Summary statistics were reported as mean ± standard error with median (range) where appropriate. Categorical variables were compared using a chi-squared test. Parametric continuous variables were compared using an independent samples t-test. Non-parametric continuous variables were compared using the Mann–Whitney U test. For comparison of more than 2 groups, statistical significance was determined using a one-way ANOVA followed by a Bonferroni multiple-group comparison test. Survival curves were evaluated using the Kaplan-Meier method. ALDH scores before and after treatment were analyzed using the two-sided paired t-test. Statistical analyses were performed using SAS version 9.2 (SAS Institute Inc., Cary, NC) and Graph-Pad Prism 5. Significance was set at P <0.05.

## Results

### ALDH^bright^ sarcoma cells display CSC properties

We first validated the CSC phenotype of ALDH^bright^ A673 Ewing’s sarcoma cells. After sorting cells by FACS into ALDH^bright^ and ALDH^dim^ sub-populations, we observed ALDH^bright^ cells were able to sustain long term survival in vitro (data not shown) and to form tumor xenografts in NSG mice (Additional file 2: Figure S2). ALDH^bright^ cells established tumors faster and were more rapidly fatal than ALDH^dim^ cells (P < 0.05). We also observed marked differences in tumor growth and volume between ALDH^bright^ and ALDH^dim^ populations on visual inspection at necropsy (Additional file 1: Figure S1B) and using T1- and T2-weighted MRI (Additional file 1: Figure S1C). In contrast, we observed CD24, CD44, and CD133 to be variably expressed in our sarcoma cell lines, and we found that these markers did not reliably correlate with the CSC phenotype (Additional file 1: Figure S1D).

### Dose dependent TKI effects in vitro

**Figure 1:**
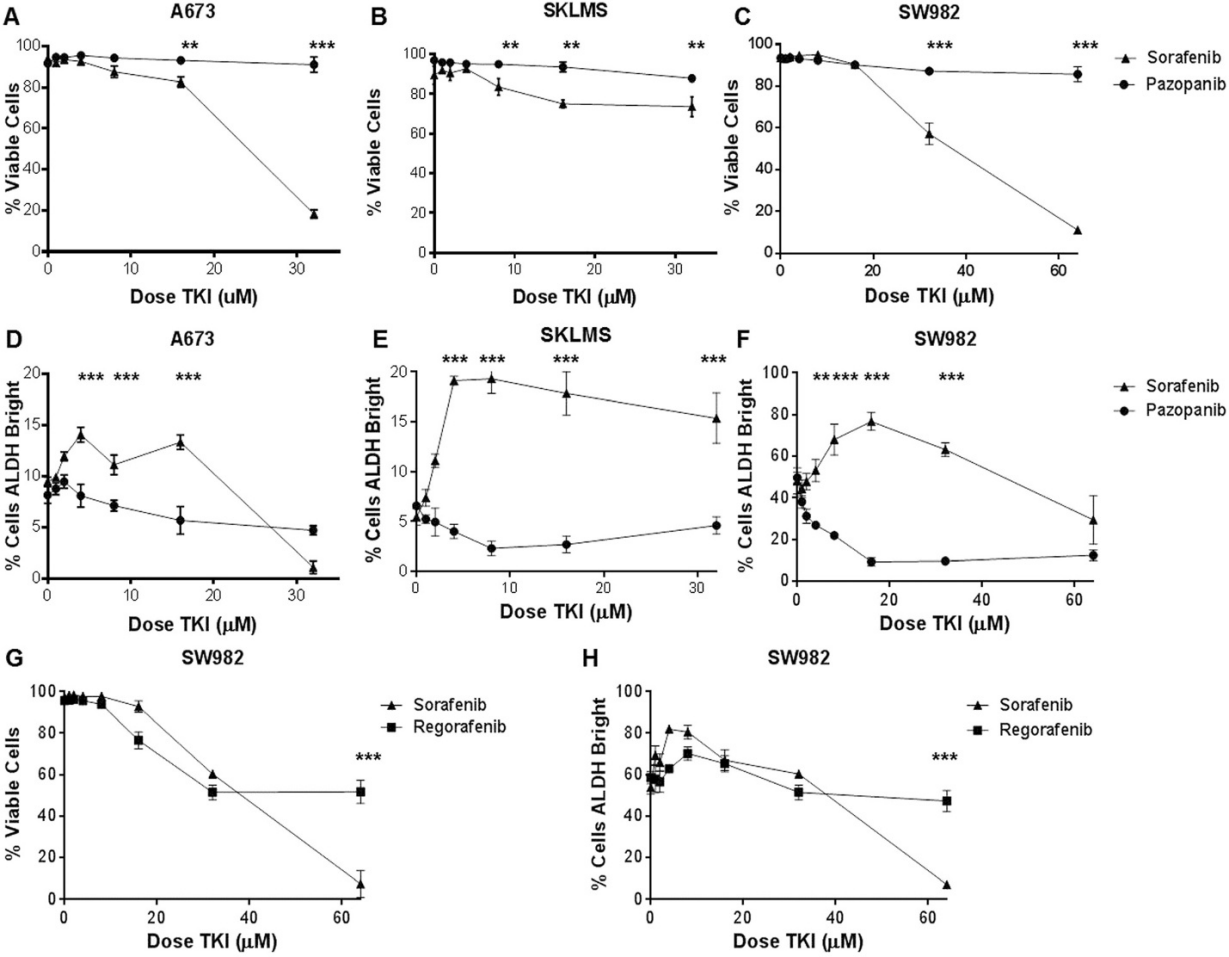
**Dose-dependent effects of TKIs on sarcoma cell line viability and CSC enrichment** ***in vitro*** **.** A673 (Ewing’s sarcoma), SK-LMS (leiomyosarcoma), and SW982 (synovial sarcoma) cells were exposed to increasing doses of sorafenib, pazopanib, and regorafenib for 18 – 24 hours. SSC^hi^ cells were then analyzed by flow cytometry using 7AAD as a cell viability stain, and ALDH expression was assessed using ALDEFLUOR™ reagents. Total cell counts and cell percentages relative to parent populations were determined. **A – C**. Cell viability was significantly inhibited in all cell lines at increasing doses of sorafenib, while no significant differences with pazopanib were observed. **D – F**. Significant enrichment of ALDH^bright^ sarcoma populations was observed in all cell lines following exposure to increasing doses of sorafenib, while negligible differences were observed with pazopanib. **G**. Sorafenib and regorafenib exert anti-proliferative effects on SW982 cells, although sorafenib is more potent than regorafenib. **H**. Sorafenib and regorafenib show similar enrichment for ALDH^bright^ CSCs. In each panel, a representative experiment of 3 replicates is shown. * P < 0.05, ** P < 0.01, and *** P < 0.001 via one-way ANOVA with Tukey’s post-test compared to dose level 0.

As depicted in Figure 1, we tested the dose-dependent effects of overnight exposure to sorafenib, pazopanib, and regorafenib on three human sarcoma cell lines. All three cell lines (Figure 1A–C) were sensitive to the cell killing effects of sorafenib at doses ranging from 8–64 μM, while pazopanib had no effect on cell viability and regorafenib demonstrated cell killing comparable to sorafenib (Figure 1G).

Concomitant with its anti-proliferative effects, sorafenib also enriched for ALDH^bright^ sarcoma CSCs in all three cell lines (Figure 1D–F). Interestingly, we observed the greatest CSC enrichment at lower doses of sorafenib than those which caused the greatest anti-viability effects. In A673 cells, for example, the ALDH^bright^ population increased from 9.5 ± 0.7% at baseline to 13.3 ± 1.3% at 4 μM (1.4X increase, P < 0.001) before dropping to 1.1 ± 1.1% at 32 μM, likely representing induction of cell death for both CSC and non-CSC population at higher doses. Similarly, sorafenib exposure enriched the ALDH^bright^ population in SK-LMS cells (Figure 1E) from 6.8 ± 2.4% at baseline to a peak of 19.3 ± 1.5% at 4 μM sorafenib (2.8X increase, P < 0.001). We also observed sorafenib enrichment for the ALDH^bright^ sub-population in SW982 cells (Figure 1 F) with the proportion rising from 48.1 ± 6.3% at 0 μM sorafenib to a peak of 76.7 ± 4.2% at 16 μM sorafenib (1.6X increase, P < 0.001). Compared to sorafenib, we did not observe any significant changes in the ALDH^bright^ populations of these cell lines following exposure to pazopanib, while regorafenib demonstrated similar CSC enrichment to sorafenib in SW982 cells (Figure 1H).

### Time dependent effects in vitro

**Figure 2:**
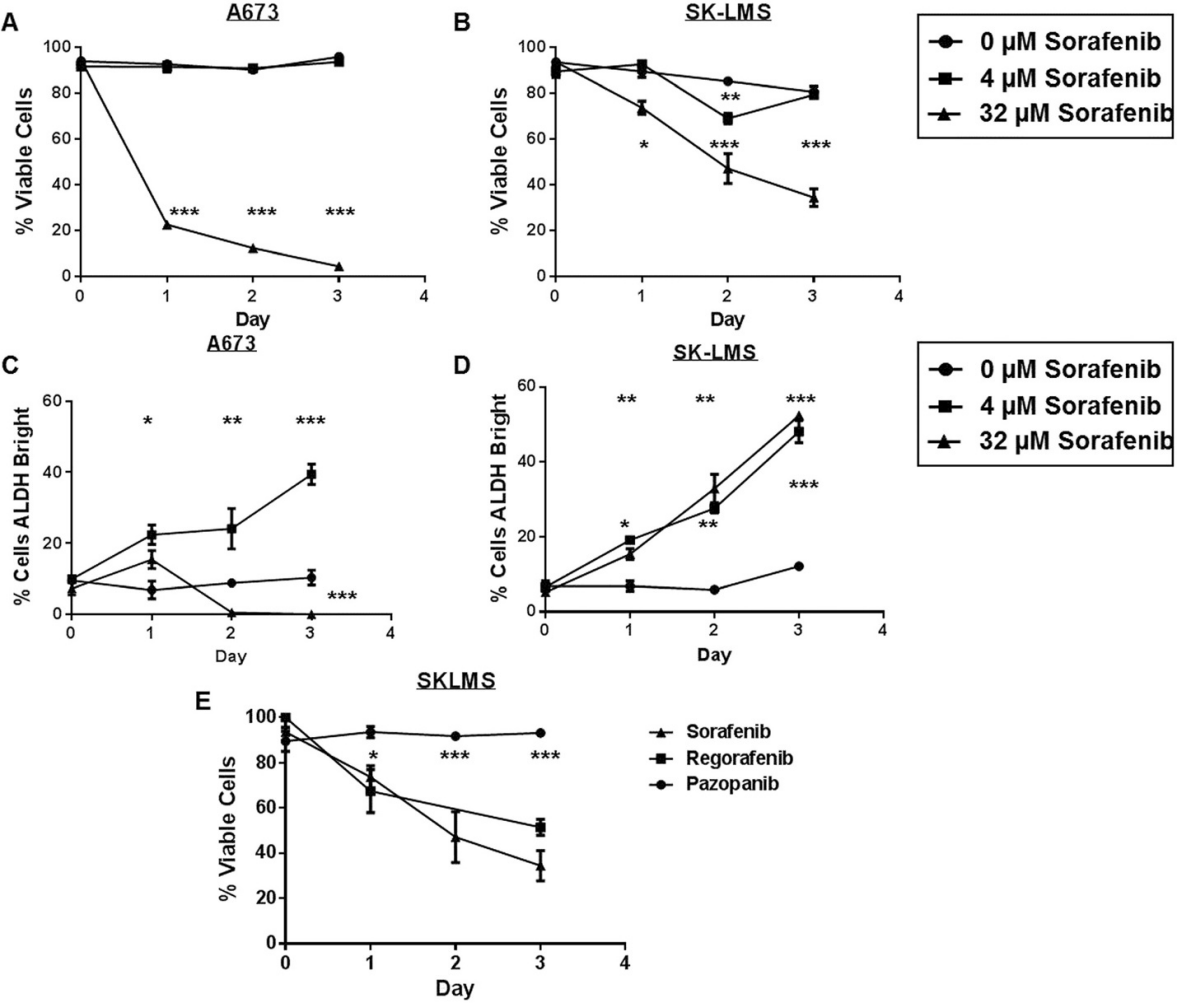
**Time-dependent effects of TKIs on sarcoma cell viability and CSC enrichment** ***in vitro*** **.** A673, SK-LMS, and SW982 cells were exposed to low- and high-dose sorafenib for 1 – 3 days. SSC^hi^ cells were then analyzed by flow cytometry using 7AAD, and ALDH expression was assessed using ALDEFLUOR™. **A**. A673 cell viability was unaffected over 3 days of culture at 4 μM sorafenib, while 32 μM sorafenib demonstrated significant reduction in cell viability over time. **B**. At 4 μM sorafenib, SK-LMS cell viability overall paralleled that of controls except for a transient decrease on day 2. In contrast, at 32 μM sorafenib, there was ongoing significant reduction in cell viability over time, although cells were less sensitive to sorafenib than A673 cells. **C**. At 4 μM sorafenib, A673 cells showed progressive significant enrichment in ALDH^bright^ sub-populations during 3 days of culture. In contrast, at 32 μM sorafenib, there is significant reduction in ALDH^bright^ sub-populations on days 2 and 3. **D**. At 4 and 32 μM sorafenib, SK-LMS ALDH^bright^ sub-populations were elevated after 1 day of sorafenib culture and continued to show enrichment in ALDH^bright^ cells on days 2 and 3. **E**. SK-LMS cell viability was unaffected over 3 days of culture at 32 μM pazopanib, while sorafenib and regorafenib demonstrated similar anti-viability effects. For each panel, a representative experiment of 3 replicates is shown. *P < 0.05, **P < 0.01, and ***P < 0.001 via one-way ANOVA with Tukey’s post-test.

We then wanted to determine if longer exposure to these TKIs increased the sensitivity of these cell lines to cell killing or CSC enrichment. At 4 μM sorafenib (Figure 2A), A673 cells remained insensitive to sorafenib, even with 3 day exposure to the drug. In contrast, culture at 32 μM sorafenib led to a cumulative loss of 87.6 ± 1.8% viable cells on day 2 (P < 0.001) and 95.5 ± 1.4% on day 3 (P < 0.001). With SK-LMS cells (Figure 2B), we also observed relatively negligible changes in viability following 3-day exposure to 4 μM sorafenib, aside from a transient decrease in viability compared to controls on day 2. Similar to A673 cells, at high dose sorafenib (32 μM), we observed progressive decreases in SK-LMS cell viability over time, decreasing to 73.7 ± 4.9% on day 1, 47.1 ± 6.5% on day 2, and 34.4 ± 6.7% on day 3, respectively (P < 0.001). The results of 3-day culture with pazopanib and regorafenib (Figure 2E) paralleled those we observed with overnight culture. There was no effect of 4 μM pazopanib on SK-LMS viability after 3 days, while 32 μM regorafenib was comparable to sorafenib with a cumulative drop in viability to 52.1 ± 3.5% (P < 0.001).

We also observed differences in the time-dependent effects of sorafenib on CSC enrichment in vitro. In A673 cells (Figure 2C), we observed a progressive enrichment in ALDH^bright^ cells with long term exposure to 4 μM sorafenib, reaching 39.4 ± 2.8% on day 3 (P < 0.001). Conversely, at 32 μM sorafenib, the percentage of ALDH^bright^ cells declined to 0.5 ± 0.9% and 0 ± 0% after 2 and 3 days of sorafenib exposure, respectively (P < 0.001). In SK-LMS cells (Figure 2D), there was a cumulative enrichment in ALDH^bright^ cells at day 2 and day 3 of exposure to 4 and 32 μM sorafenib, respectively, whereas there was no significant change in the percentage of ALDH^bright^ cells over 3 days among the vehicle-treated controls.

From these data, we concluded that longer exposure to low dose sorafenib led to persistent CSC enrichment without any increase in anti-proliferative effects, suggesting a possible mechanism of resistance to the drug at lower doses. High dose sorafenib was effective at inducing cell death in both CSC and non-CSC populations in A673 cells, but in SK-LMS cells there was less sensitivity to the drug and a corresponding progressive enrichment in ALDH^bright^ cells, also suggesting that enrichment in CSC populations may correlate with drug resistance.

### Sorafenib exerts anti-proliferative effects *in vivo*while enriching for sarcoma CSCs

**Figure 3:**
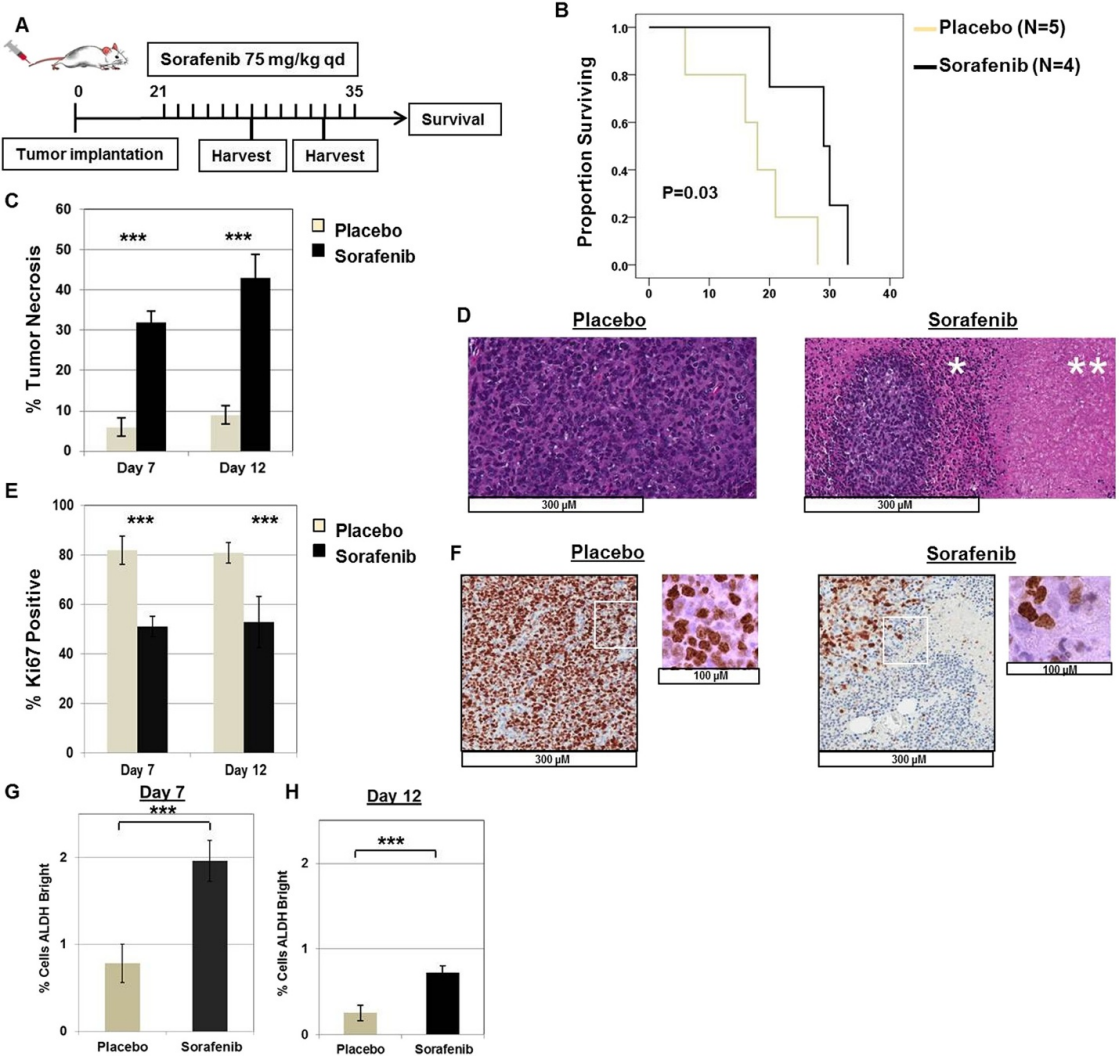
**Effects of sorafenib on A673 xenografts in vivo. A**. Schema depicts experimental design *in vivo*. Sarcoma cells were infused by tail vein on day 0. Xenografts were allowed to develop for 21 days, and mice were then randomized to i.p. injections of placebo versus sorafenib (75 mg/kg) x 21 days (or until tumor harvest). **B**. Mice treated with sorafenib survived significantly longer than placebo-treated mice (median survival post-treatment 29 days vs. 18 days, P = 0.03 by log-rank test). **C**. Sorafenib-treated metastatic lung tumors demonstrated significantly greater tumor necrosis than placebo-treated tumors at indicated time points (P < 0.001 by paired t-test at both time points). **D**. Representative images of placebo- and sorafenib-treated tumors stained with hematoxylin and eosin (H&E) on treatment day 12. Placebo-treated tumors remain highly cellular and mitotically active with minimal tumor necrosis, while sorafenib-treated tumors show necrosis (**) and inflammation (*) surrounding areas of viable tumor. **E**. Sorafenib-treated metastatic lung tumors demonstrated significantly decreased numbers of Ki67 positive cells than placebo-treated tumors at day 7 and day 12 of treatment (P < 0.001 by paired t-test at both time points). **F**. Representative images of placebo- and sorafenib-treated tumors stained for Ki67 on treatment day 7. Non-necrotic, viable areas of sorafenib-treated tumors demonstrate fewer proliferating cells than placebo-treated tumors. Boxed areas are depicted at higher magnification. **G – H**. Metastatic lung tumors harvested at indicated time points demonstrate statistically greater ALDH^bright^ CSCs than placebo-treated controls (P < 0.001 by paired t-test). For all panels, representative data from 2 – 3 experiments are shown.

We then tested the effects of sorafenib in an in vivo model. Injection of A673 cells into NSG mice by tail vein reproducibly produced lung and liver metastases after 21 days. We then treated mice in groups of 4–5 with daily injections of i.p. sorafenib or vehicle until tissues were harvested or survival studies were completed (Figure 3A). Previous studies have observed in vivo effects of sorafenib at doses ranging from 30–100 mg/kg [20, 31]. Although we observed a linear relationship between sorafenib dose in vivo and tumor growth delay, 100 mg/kg was toxic to approximately 10% of animals. Therefore, we opted to use 75 mg/kg as the in vivo dose of sorafenib.

As shown in Figure 3B, we observed that sorafenib prolonged survival in vivo by a median of 11 days from 18 days post-initiation of treatment to 29 days (P = 0.03). Histological evaluation of tumors after sorafenib treatment revealed a statistically significant increase in tumor necrosis (Figure 3C). On day 7 post-treatment, percent tumor necrosis was 6.0 ± 2.2% in placebo-treated animals vs. 32.0 ± 2.7% in sorafenib-treated animals (P < 0.001). There was also a significant increase in percent tumor necrosis in sorafenib-treated animals on day 12 post-treatment (P < 0.001). Figure3D shows representative micrographs of tumor histology from placebo-treated and sorafenib-treated animals, respectively.

As shown in Figure 3E, tumor cell proliferation as measured by Ki-67 staining was significantly higher in placebo-treated than sorafenib-treated animals. In placebo-treated animals, the percentage of Ki-67 positive cells was 82 ± 6% and 81 ± 4% on days 7 and 12 post-treatment, respectively. In contrast, in sorafenib-treated animals, the percentage of Ki-67 positive cells was 51 ± 4% and 53 ± 10% on days 7 and 12 post-treatment, respectively (P < 0.001). Non-viable, necrotic areas were excluded from the calculation of Ki-67 staining.

We then evaluated A673 xenografts for changes in ALDH^bright^ populations after sorafenib and placebo treatment. On day 7 post-treatment (Figure 3G), we observed the ALDH^bright^ sub-population to be significantly higher in sorafenib-treated tumors than placebo-treated tumors (P < 0.001). Similarly, on day 12 post-treatment (Figure 3H), we observed the ALDH^bright^ sub-population to be higher in the sorafenib-treated animals than placebo-treated ones, 0.72 ± 0.08% vs. 0.25 ± 0.09%, respectively (P < 0.001). Although the absolute differences in ALDH^bright^ sub-populations between placebo and sorafenib-treated animals were relatively modest, these differences nevertheless represented 2.5–2.9 fold enrichment in the CSC population at both time points.

Based on these data, we concluded that sorafenib exerts anti-proliferative effects in vivo while simultaneously enriching for CSCs, suggesting a preferential anti-proliferative effect on the non-CSCs.

### Sorafenib is cytotoxic to human primary sarcomas ex vivo but enriches for sarcoma CSCs

**Figure 4:**
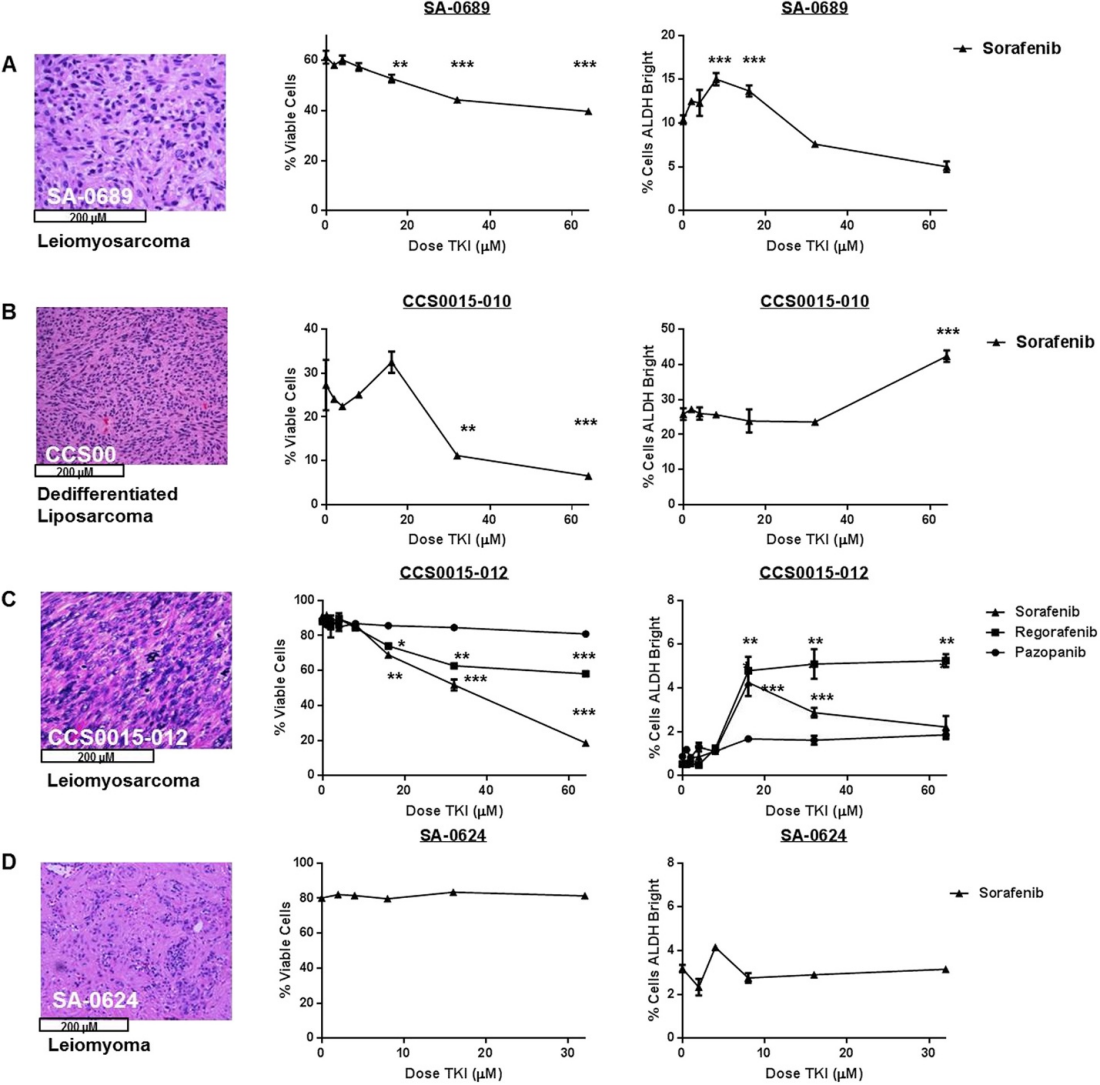
**Effects of TKIs on viability and CSC enrichment in short term ex vivo culture of resected clinical sarcoma tumors. A.** (Left) High power view shows moderately differentiated recurrent renal vein leiomyosarcoma from patient SA-0689. Freshly digested tumor cells were exposed to increasing doses of sorafenib for 16 – 18 hours and then analyzed by flow cytometry for viability and ALDH expression using 7AAD and ALDEFLUOR™, respectively. (Middle) Ex vivo tumor cell viability was significantly inhibited at ≥16 μM of sorafenib. (Right) Significant enrichment of ALDH^bright^ sarcoma cells was observed at sorafenib doses of 8 and 16 μM. **B.** (Left) High power view shows highly cellular, non-lipogenic dedifferentiated liposarcoma from patient CCS0015-010, confirmed by MDM2 overexpression. (Middle) Sorafenib significantly decreases ex vivo tumor viability at 32 and 64 μM, while simultaneously enriching for ALDH^bright^ sarcoma cells doses ≥64 μM (Right). **C**. (Left) High power view shows poorly-differentiated high grade leiomyosarcoma from patient CCS0015-012 exposed to sorafenib, regorafenib, and pazopanib. (Middle) Cell viability was significantly inhibited at ≥16 μM of sorafenib and regorafenib, while no significant differences were observed following pazopanib exposure. (Right) Significant enrichment of ALDH^bright^ sarcoma cells was observed following sorafenib and regorafenib exposure, while negligible differences were observed with pazopanib. **D**. (Left) High power view shows benign leiomyoma from patient SA-0624. No significant effects on cell viability (Middle) nor on ALDH^bright^ CSCs (Right) were observed following exposure to increasing doses of sorafenib. All experiments were performed in triplicate. *P < 0.05, **P < 0.01, and *** P < 0.001 via one-way ANOVA with Tukey’s post-test compared to dose level 0.

We then analyzed the effects of TKIs on tumor cells freshly isolated from STS specimens (and benign soft tissue tumors) obtained at the time of surgical resection. There was marked patient to patient heterogeneity of tumor cells and the percentage of ALDH^bright^ cells detected at baseline (Figure 4A–D). Leiomyosarcoma cells from patient SA-0689 (Figure 4A) decreased in viability from 61.3 ± 2.6% at baseline to 44.3 ± 0.2% and 39.7 ± 0.4% at 32 μM and 64 μM sorafenib, respectively (P < 0.001) with a corresponding enrichment in ALDH^bright^ cells from 10.4 ± 0.5% at baseline to a peak of 15.0 ± 0.7% at 8 μM sorafenib (P < 0.001). For patient CCS0015-010 (Figure 4B), sorafenib decreased the viability of dedifferentiated liposarcoma cells from 26.9 ± 0.5% at baseline to 11.2 ± 0.6% at 32 μM sorafenib (P < 0.01) and 6.6 ± 0.3% at 64 μM sorafenib (P < 0.001), respectively. There was simultaneous enrichment for the ALDH^bright^ sub-population which increased from 24.9 ± 0.8% at baseline to 42.4 ± 1.6% at 64 μM sorafenib (P < 0.001).

Leiomyosarcoma cells from patient CCS0015-012 (Figure 4B) were highly sensitive to sorafenib ex vivo with a dose-dependent decrease in viability from 90.5 ± 0.3% at baseline to 18.2 ± 1.3% at 64 μM sorafenib (P < 0.001). These tumor cells demonstrated intermediate sensitivity to regorafenib, while there was no significant change in viability following pazopanib exposure. Simultaneously, there was a corresponding enrichment in CCS0015-012 ALDH^bright^ cells following exposure to both sorafenib and regorafenib. Interestingly, similar to our in vitro experiments, CSC enrichment following sorafenib peaked at lower doses of sorafenib (16 μM) and then dropped at higher doses whereas CSC enrichment following regorafenib remained elevated at higher doses. We then tested a benign leiomyoma (Figure 4D) to ascertain whether some of these effects could be attributed to the ex vivo digestion process. We observed no significant change in cell viability or the ALDH^bright^ population following sorafenib exposure.

Despite the variability between patient samples, we concluded from these data that sorafenib was directly cytotoxic to human primary sarcomas ex vivo with a corresponding increase in ALDH^bright^ cells, and our ex vivo results with pazopanib and regorafenib correlated with our results in vitro.

### Preoperative sorafenib enriches for CSCs in clinical sarcoma specimens

We then analyzed ALDH1 staining by IHC from archived specimens of STS patients. We created a TMA using replicate cores of tumor tissue obtained from STS patients previously treated with neoadjuvant sorafenib and conformal RT on a Phase I clinical trial for patients with locally advanced disease amenable to treatment with curative intent (Clinical Trial Information: NCT#00805727/UCDCC#216) [24]. Patients underwent a core biopsy to establish the diagnosis of STS (pre-treatment) followed by 5–6 weeks of preoperative treatment with sorafenib at one of two doses (200 mg bid and 200/400 daily) with concurrent RT. Surgical resection was performed 4–6 weeks following completion of neoadjuvant therapy.

**Figure 5:**
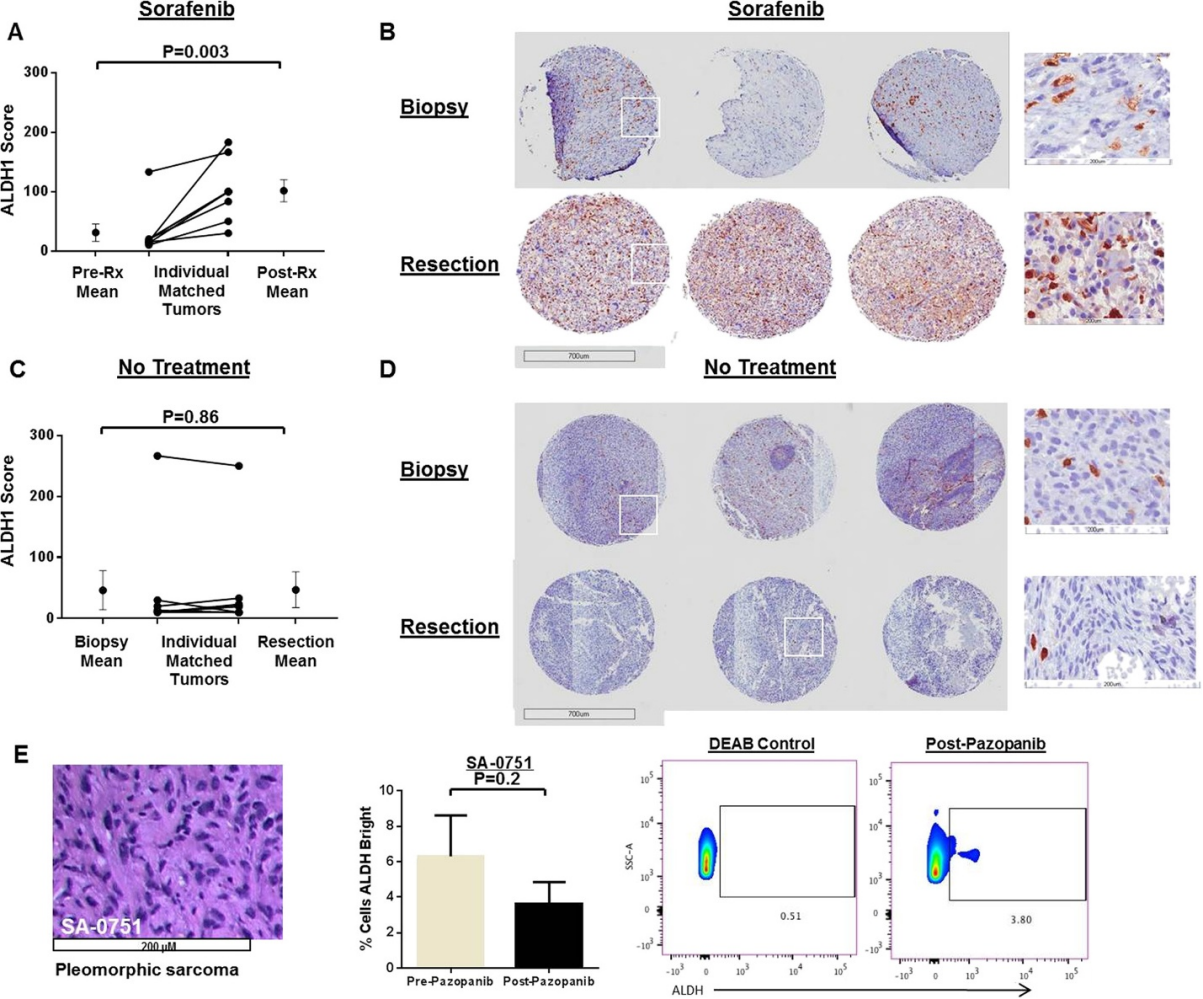
**ALDH1 expression in clinical specimens of sarcoma patients treated with preoperative sorafenib or pazopanib prior to surgical resection. A**. Increases in ALDH1 staining intensity among soft tissue sarcoma patients treated with neoadjuvant sorafenib and conformal radiotherapy on a Phase I trial (Clinical Trial Information: NCT#00805727/UCDCC#216). **B**. Representative photomicrographs of ALDH1 expression from a patient pre- (top) and post-treatment (bottom) evaluated on tissue microarray. Three cores per patient were analyzed. Boxed areas are depicted at higher magnification. **C**. Negligible effects in ALDH1 staining intensity among soft tissue sarcoma patients sarcoma ineligible for NCT#00805727/UCDCC#216 who underwent diagnostic biopsy followed by definitive surgical resection without preoperative therapy. **D**. Representative photomicrographs of ALDH1 expression from a patient pre- (top) and post-treatment (bottom) from a tissue microarray created from formalin-fixed, paraffin-embedded specimens of patients with soft tissue sarcoma treated with a biopsy followed by definitive surgical resection without preoperative therapy. Three cores per patient were analyzed. Boxed areas are depicted at higher magnification. **E**. High power view shows high grade undifferentiated pleomorphic sarcoma from patient SA-0751 treated clinically with pazopanib for metastatic sarcoma. (Middle) Flow cytometry reveals no significant difference in ALDH^bright^ cells from fresh tissue evaluated before and after pazopanib. (Right) Representative flow cytometry plots of ALDH expression are shown. ALDH scores before and after treatment were analyzed using the two-sided paired t-test. All experiments were performed in triplicate.

Using matched patient tissue before and after sorafenib therapy, we observed increases in the ALDH1 score among all patients (Figure 5A). Moreover, the mean ALDH1 score increased significantly from 31 ± 14 pre-treatment to 101 ± 18 post-treatment (P = 0.003). We then evaluated ALDH1 staining intensity in a control cohort of STS patients who declined participation or were ineligible for NCT#00805727/UCDCC#216. These patients underwent diagnostic biopsy followed by definitive surgical resection without preoperative therapy. As shown in Figure 5C, using matched tissue from these patients, we observed no difference in the mean ALDH1 score from tumor tissue obtained at biopsy versus tissue obtained at surgical resection (biopsy mean ALDH1 score 46 ± 34 vs. resection mean score 44 ± 27, P = 0.86).

We then examined paired samples of tissue from a patient with metastatic STS who was treated with pazopanib and underwent surgical resection before and after treatment (Figure 5E). There was no significant difference in the percentage of ALDH^bright^ cells present in the specimen after pazopanib treatment, providing additional evidence from STS patients that sorafenib enriches for ALDH^bright^ sarcoma CSCs while pazopanib does not.

## Discussion

Accumulating evidence suggests that CSCs exist as a sub-population of quiescent cells within the dominant tumor bulk of heterogeneous tumor cells [1, 12]. These typically dormant cells are considered resistant to standard anti-cancer therapies such as chemotherapy and RT, and they appear capable of self-renewal and differentiation [5, 19, 32, 33], suggesting that CSCs are responsible for tumor repopulation after bulk tumor has been destroyed [3].

Numerous studies have focused on characterizing the behavior and phenotype of CSCs. Much attention has been devoted to demonstrating that the expression of cell surface markers, such as CD24, CD44, and CD133, and the activity of the intracellular enzyme ALDH consistently predict the CSC phenotype. In addition, the presence of CSCs, in general, and ALDH expression, in particular, has been shown to predict worse prognosis in numerous human cancers, such as breast, prostate, and kidney [13, 14, 17]. Despite this attention to the significance of CSCs, relatively few studies have examined the differences in anti-proliferative versus anti-angiogenic therapy on the evolution of CSC subpopulations over time in cell culture, xenograft models, or clinical specimens, particularly for STS.

In this study, we demonstrate that sorafenib and regorafenib, but not pazopanib, exert significant anti-proliferative effects while simultaneously enriching for CSCs in multiple models of STS, including primary sarcoma cells freshly derived from surgical specimens. In addition, we observe enrichment of ALDH1-stained cells in matched tumor-specimens obtained before and after neoadjuvant therapy with sorafenib and RT, whereas there was no evidence of CSC enrichment following clinical treatment with pazopanib. Although CSCs are widely believed to be resistant to conventional cytotoxic therapies, such as chemotherapy and RT, there are relatively few studies which demonstrate similar effects with anti-proliferative TKIs, such as sorafenib. There are also limited data evaluating the differences on CSC enrichment between various TKIs.

Our study reinforces the concept that anti-proliferative therapies enrich for the CSC population in solid malignancies and that anti-proliferative TKIs may exert distinct anti-tumor strategies than anti-angiogenic ones. A major strength of our study is the substantial amount of data obtained from STS patients, including fresh STS specimens procured at the time of surgical resection as well as retrospectively from archived tumor specimens. These data emphasize the translational relevance of our work which suggests that CSC enrichment following TKI therapy with sorafenib may be a mechanism of tumor resistance. Consequently, we hypothesize that sustained and durable anti-sarcoma therapies will require concomitant targeting of CSCs.

These data also reinforce the broader concept that standard anti-proliferative therapies, including TKIs, target the proliferating non-CSCs while sparing, or possibly even promoting, the repopulation of CSCs. Our data also support the hypothesis that CSCs are a mechanism of resistance to standard anti-proliferative therapies since elimination of non-CSCs parallels the enrichment of CSCs.

Although we demonstrate a consistent pattern for the effects on sorafenib (distinct from pazopanib) on STS in diverse pre-clinical and human models, it is important to acknowledge several limitations of our study. Although the majority of STS subtypes share a common mesenchymal origin, STS are a heterogenous group of malignancies, and there is clearly a variation in ALDH expression from subtype to subtype as well as depending on the cell culture conditions. Despite this variability in ALDH expression, we focused on ALDH activity as a CSC marker since we could validate the phenotype of ALDH^bright^ CSCs. In contrast, similar to Chen et al., we observed that CD133 and CD44 did not reliably validate the CSC phenotype [34]. However, the variability of marker expression among CSCs depending on culture conditions as well as tumor histology does imply that we may have to adapt the techniques necessary to identify and target sarcoma CSCs by subtype and reinforces the concept of some critics that ALDH and other cell surface molecules only correlate with the CSC phenotype rather than causally mediate it [12, 35].

In addition, the majority of our pre-clinical data was obtained from a Ewing’s sarcoma cell line (A673). This STS subtype is known to share many phenotypic properties with neuro-ectodermal cells [36]. Consequently, it is possible that our results with this cell line are biased by the overlap of A673 cells with neural progenitor tissue, which are known to harbor pluripotent stem cells [37]. Finally, our data from archived STS specimens treated on a neoadjuvant clinical protocol including sorafenib is confounded by the addition of RT to the treatment regimen. Although there is significant enrichment after sorafenib/RT compared to no treatment, it is conceivable that some, or the majority, of these effects is secondary to RT or the combination of sorafenib and RT, rather than sorafenib alone.

## Conclusion

In summary, we demonstrate that in diverse sarcoma models, including extensive clinical sarcoma specimens, sorafenib exerts significant anti-proliferative effects while simultaneously enriching for CSCs, and pazopanib does not. These data indicate that TKIs have differential effects on CSC populations depending on their mechanism of action. Taken together, our results suggest that CSC enrichment following anti-proliferative TKI therapy is an apparent mechanism of tumor resistance. Therefore, sustained anti-sarcoma therapies may require concomitant targeting of CSCs.

## Electronic supplementary material

Additional file 1: **Representative Flow Cytometry Plots of ALDH Expression A.** SW982 cells in vitro are shown. **(Left)** Diethylaminobenzaldehyde (DEAB), a specific inhibitor of ALDH, is used to control for background fluorescence. **(Middle)** Vehicle control. **(Right)** Sorafenib 16 uM. **B.** A673 in vivo tumors harvested on treatment day 7 are shown. **(Left)** DEAB background fluorescence. **(Middle)** Placebo-treated controls. **(Right)** Sorafenib 75 mg/kg. (JPG 67 KB)

Additional file 2: Figure S1: Validation of ALDH as a CSC marker in A673 sarcoma cells. **A.** A673 cells were sorted by flow cytometry into ALDH^bright^ and ALDH^dim^ populations. 2 × 10^5^ purified cells were implanted subcutaneously into contralateral flanks of NSG mice (N = 4) and allowed to grow. ALDH^bright^ cells established tumors faster and were more rapidly fatal. *P < 0.05. **B.** Representative photograph showing difference in tumor formation between ALDH^bright^ and ALDH^dim^ A673 sarcoma cells sorted by flow cytometry and implanted subcutaneously in NSG mice. **C.** Representative T1- and T2-weighted MRI images demonstrating difference in tumor formation between ALDH^bright^ and ALDH^dim^ A673 sarcoma cells sorted by flow cytometry and implanted subcutaneously in NSG mice. **D.** Expression of CSC cell surface markers and ALDH in representative STS cell lines. (JPG 73 KB)

## Abbreviations

CSCs: Cancer stem cells
STS: Soft tissue sarcoma
ALDH: Aldehyde dehydrogenase
TMA: Tissue microarray
RT: Radiotherapy
TKIs: Tyrosine kinase inhibitors
i.p.: Intraperitoneal
DEAB: Diethylaminobenzaldehyde
FACS: Fluorescence-activated cell sorting
H&E: Hematoxylin and eosin.
