# Supplementary figures and images for: Anti-proliferative but not anti-angiogenic tyrosine kinase inhibitors enrich for cancer stem cells in soft tissue sarcoma
Source: BMC Cancer. 2014 Oct 10;14:756. doi: 10.1186/1471-2407-14-756 (PMC4200119; doi:10.1186/1471-2407-14-756)

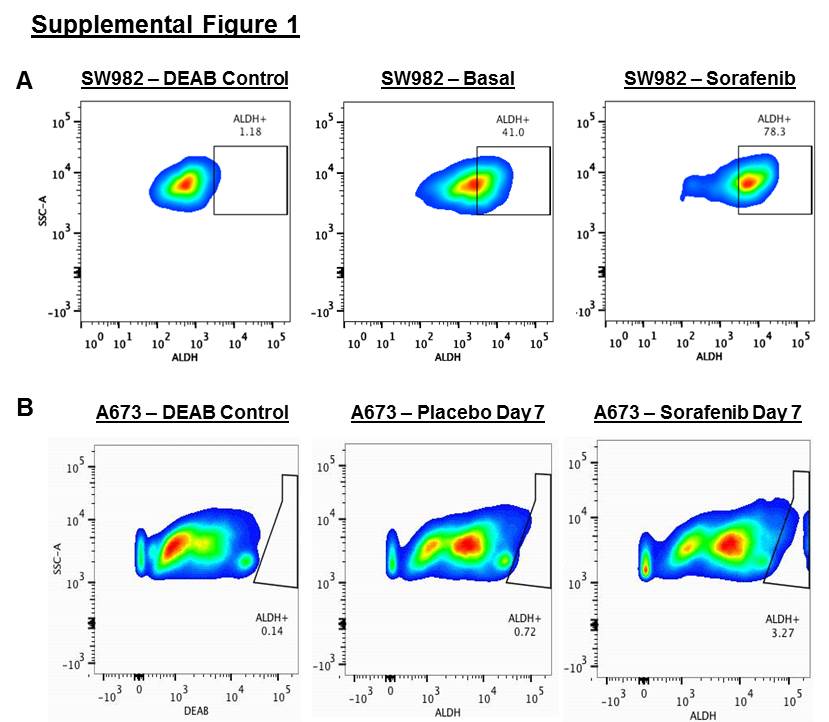

Supplement: Supplementary file 1 — Additional file 1: Representative Flow Cytometry Plots of ALDH Expression A. SW982 cells in vitro are shown. (Left) Diethylaminobenzaldehyde (DEAB), a specific inhibitor of ALDH, is used to control for background fluorescence. (Middle) Vehicle control. (Right) Sorafenib 16 uM. B. A673 in vivo tumors harvested on treatment day 7 are shown. (Left) DEAB background fluorescence. (Middle) Placebo-treated controls. (Right) Sorafenib 75 mg/kg. (JPG 67 KB) [file 12885_2014_4939_MOESM1_ESM.jpg]

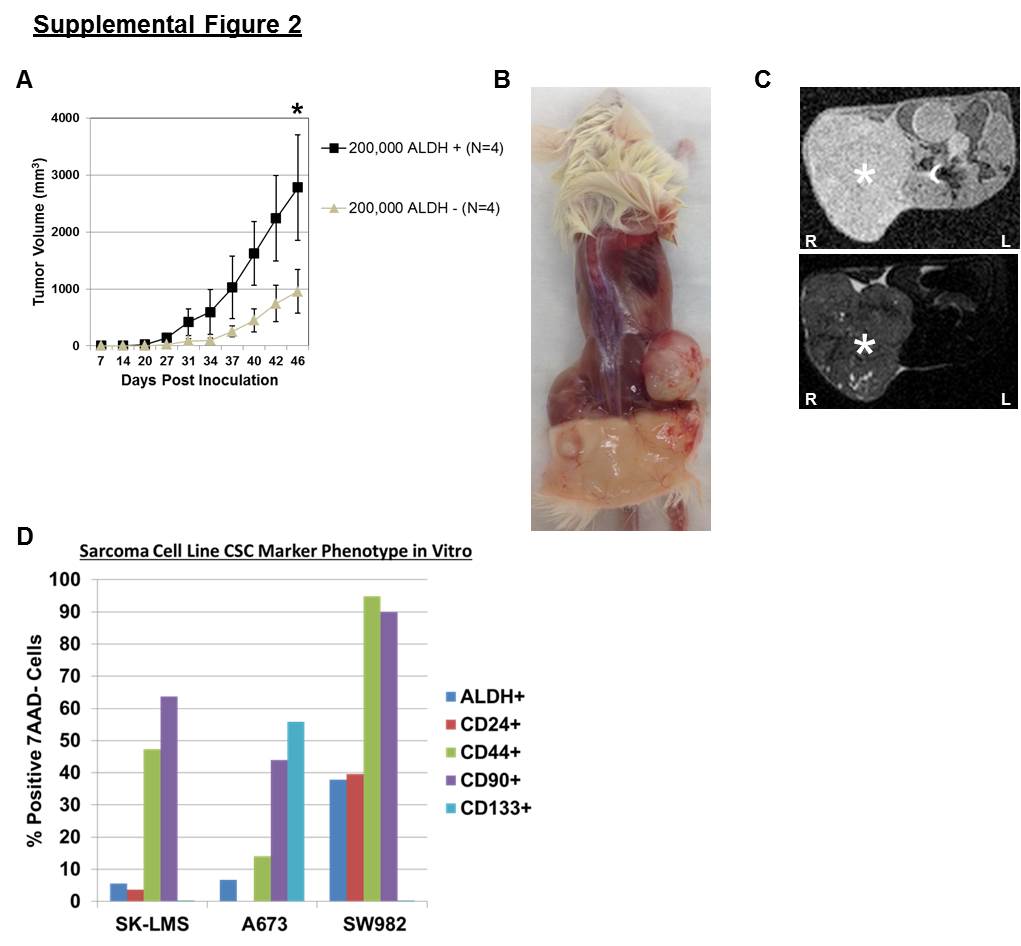

Supplement: Supplementary file 2 — Additional file 2: Figure S1: Validation of ALDH as a CSC marker in A673 sarcoma cells. A. A673 cells were sorted by flow cytometry into ALDHbright and ALDHdim populations. 2 × 10^5^ purified cells were implanted subcutaneously into contralateral flanks of NSG mice (N = 4) and allowed to grow. ALDHbright cells established tumors faster and were more rapidly fatal. *P < 0.05. B. Representative photograph showing difference in tumor formation between ALDHbright and ALDHdim A673 sarcoma cells sorted by flow cytometry and implanted subcutaneously in NSG mice. C. Representative T1- and T2-weighted MRI images demonstrating difference in tumor formation between ALDHbright and ALDHdim A673 sarcoma cells sorted by flow cytometry and implanted subcutaneously in NSG mice. D. Expression of CSC cell surface markers and ALDH in representative STS cell lines. (JPG 73 KB) [file 12885_2014_4939_MOESM2_ESM.jpg]
